# M^2^AML: Metric-Based Model-Agnostic Meta-Learning for Few-Shot Classification

**DOI:** 10.3390/e28050484

**Published:** 2026-04-23

**Authors:** Xiaoming Han, Dianxi Shi, Zhen Wang, Shaowu Yang

**Affiliations:** 1 College of Computer Science and Technology, National University of Defense Technology, Changsha 410000, China; hanxiaoming22@nudt.edu.cn (X.H.); shaowu.yang@nudt.edu.cn (S.Y.); 2 School of Electronics and Information, Xi’an Polytechnic University, Xi’an 710048, China; frozenzhencola@163.com

**Keywords:** few-shot learning, meta-learning, MAML, prototypical networks

## Abstract

Model-Agnostic Meta-Learning (MAML) and Prototypical Networks (ProtoNet) establish the foundational paradigms for few-shot classification. However, MAML suffers from optimization instability caused by reconstructing classification boundaries for every new task. Conversely, ProtoNet lacks the internal mathematical capacity necessary for task-specific parameter adaptation under domain shifts. To reconcile these structural limitations, we introduce Metric-based Model-Agnostic Meta-Learning (M^2^AML). By completely excising the parameterized classification layer from the episodic adaptation sequence, our framework replaces traditional inner-loop classification with a dynamic self-exclusive geometric similarity metric. Substituting functional mappings with spatial distance optimizations efficiently resolves evaluation conflicts, thereby establishing perfectly synchronized inner and outer learning rates alongside substantially accelerated adaptation steps. Extensive experiments across *mini*-ImageNet, *tiered*-ImageNet, and CIFAR-FS validate our approach against a comprehensive array of established algorithms. To ensure strictly fair comparative evaluations, we meticulously reproduce the MAML, ProtoNet, and Proto-MAML baselines. Empirical results demonstrate that M^2^AML achieves state-of-the-art performance across most evaluation settings, delivering absolute accuracy improvements ranging from 0.1% to 2.1% over existing leading models.

## 1. Introduction

Contemporary deep learning achieves remarkable success with massive datasets [1,2,3]; yet, this heavy data dependency restricts applicability in specialized domains where large-scale collection is impractical. Few-shot classification explicitly addresses this bottleneck by requiring models to generalize from extremely limited annotated data [4,5]. This paradigm effectively resolves data scarcity constraints across diverse disciplines ranging from ecological species range estimation [6] and computational drug discovery [7] to sparse time series forecasting [8].

To mathematically construct this remarkable generalization capability, contemporary research predominantly explores gradient-based meta-learning and metric-based representation learning [9]. Gradient-based architectures, exemplified by Model-Agnostic Meta-Learning (MAML), focus on discovering a universally adaptable global initialization [10,11,12,13]. This strategic initialization enables rapid convergence to new task distributions through targeted gradient descent steps, providing dynamic representational capacity to explicitly handle substantial domain shifts. Conversely, metric-based frameworks, such as Prototypical Networks (ProtoNet) [14,15,16,17], map input samples into a highly discriminative stationary embedding space. Within this geometric space, classification relies entirely on computing spatial similarities to aggregated class centroids without requiring task-specific parameter fine-tuning. This fundamental approach inherently provides highly stable training dynamics and robust resistance to overparameterization under extreme data sparsity.

Despite their respective advantages, both paradigms exhibit fundamental structural limitations that significantly impact operational efficiency and performance, as detailed in Table 1. The standard MAML architecture must explicitly reconstruct a randomly initialized linear classification boundary for every new task during the inner adaptation loop. This mechanism introduces severe optimization instability and distorts early gradients, forcing the use of complex configurations with separate learning rates for inner adaptation and outer updates. Consequently, this intricate bi-level optimization results in notoriously slow meta-training convergence and sluggish inference speeds. In contrast, purely metric-based frameworks like ProtoNet eliminate the inner adaptation loop entirely, which affords them remarkably fast training and inference times alongside a complete absence of inner learning rate hyperparameter tuning. However, since they freeze their feature extraction backbones during the test phase inference, they are deprived of the internal mathematical capacity necessary for task-specific parameter adaptation under substantial domain shifts, yielding sub-optimal accuracy, particularly in one-shot scenarios.

To definitively resolve these structural requirements and bridge the gap between gradient-based and metric-based methodologies, Proto-MAML attempted to integrate both paradigms by utilizing geometric prototypes to initialize the linear classification layer [18]. However, this approach merely alters the initial state of the linear classifier and fundamentally preserves the identical gradient optimization bottlenecks inherent to MAML without actually resolving the foundational instability. In response to these persistent limitations, this research introduces a novel geometric framework titled Metric-based Model-Agnostic Meta-Learning, denoted as M^2^AML. The proposed architecture completely excises the parameterized classification layer from the episodic adaptation sequence. Instead of engaging in traditional inner loop linear classification, the framework dynamically calculates spatial geometric similarities between support samples and adaptive class prototypes. Replacing functional linear mappings with spatial distance optimizations efficiently resolves historical evaluation conflicts. This geometric substitution establishes perfectly synchronized inner and outer learning rates while substantially accelerating the underlying task adaptation steps. Furthermore, directly applying standard aggregated prototypes within the inner optimization loop creates a fatal self-matching optimization trap. Because support samples simultaneously act as evaluation queries and formulate cluster boundaries, calculating similarities without sample isolation minimizes self-inclusive distances rather than abstracting robust geometric metrics. To prevent this mathematical collapse, the framework incorporates a dynamic self-exclusive computation mechanism. This abstract algorithmic structure rigorously masks local instances from their respective class-prototype calculations, thereby guaranteeing unbiased gradient generation and consistent mathematical stability. To rigorously validate the efficacy of the proposed architecture, comprehensive empirical evaluations are conducted across three standardized few-shot classification benchmarks, specifically *mini*-ImageNet, *tiered*-ImageNet, and CIFAR-FS. In pursuit of absolute empirical fairness, classical baselines, including MAML, ProtoNet, and Proto-MAML, are rigorously reproduced under identical parameter configurations. Empirical investigations explicitly demonstrate that the proposed structural design naturally achieves optimal convergence utilizing a completely synchronized learning rate for both optimization loops, thereby fundamentally eliminating the historically complex necessity to independently tune distinct inner and outer hyperparameters. Ultimately, quantitative findings prove that the M^2^AML framework consistently achieves state-of-the-art performance across most evaluation settings, delivering absolute accuracy improvements ranging from 0.1% to 2.1% over existing leading models.

The primary contributions of this research organically emerge from these analytical efforts and are formally summarized as follows:

- The formulation of M^2^AML, a novel hybrid meta-learning architecture that completely excises parameterized classification layers, thereby computationally eliminating the optimization oscillation mathematically inherent in standard inner loop adaptations.
- The design of an unbiased self-exclusive geometric prototype validation sequence synergized with robust batch normalization stabilization, which successfully enables perfectly synchronized learning rates across iterative episodic adaptation and global structural updates.
- Comprehensive empirical validation demonstrating that the proposed methodology consistently achieves state-of-the-art accuracy across diverse evaluation settings and practically resolves the structural dichotomy between metric efficiency and gradient adaptation within the investigated domains.

The remainder of this manuscript is organized as follows. Section 2 contextualizes the proposed framework within existing literature. Section 3 delineates the problem formulation and the algorithmic pipeline of the M^2^AML architecture. Section 4 presents comprehensive experimental evaluations and comparative analyses. Finally, Section 5 synthesizes the core findings and outlines future directions.

## 2. Related Work

This section reviews prior studies most relevant to the proposed framework from three complementary perspectives. It first summarizes metric-based few-shot learning, then examines gradient-based model-agnostic meta-learning, and finally discusses integration strategies to clarify the methodological position of M^2^AML.

### 2.1. Metric-Based Few-Shot Learning Mechanisms

Metric-based few-shot learning builds embedding spaces that map semantically similar instances to nearby locations. Prototypical Networks [14] establish this paradigm by classifying query samples through Euclidean distances to class mean prototypes computed from sparse support examples. This formulation creates robust decision boundaries without learnable classification heads and therefore reduces the risk of overparameterization. Building on this foundation, Matching Networks [19] employ continuous attention across the full support set rather than relying only on aggregated centroids. TADAM [20] introduces task-dependent conditioning, scalar metric adjustments, and auxiliary co-training procedures to improve distance-based evaluations. DeepEMD [21] formulates classification as an optimal transport problem through Earth Mover distance and a cross-reference mechanism that suppresses background interference while preserving local structural correspondence. FEAT [16] improves discriminative representations by applying transformer modules to model holistic interactions among support samples. Meta-baseline [17] shows that targeted meta-learning updates on a globally pretrained representation manifold can further strengthen metric inference. FewTure [22] partitions images into local patches with vision transformers and combines masked modeling with online optimization to capture informative fine-grained semantics. Overall, metric methods benefit from clear geometric inductive biases and stable training dynamics because they avoid task-specific classifier optimization. They also support direct inference on novel categories without fine-tuning. However, because the backbone is fixed at test time, purely metric approaches often lack the parameter adaptation capacity needed under substantial domain shift.

### 2.2. Gradient-Based Model-Agnostic Meta-Learning

Gradient-based meta-learning addresses task generalization through direct parameter optimization. The foundational Model-Agnostic Meta-Learning approach [10] learns a global initialization that can adapt rapidly with only a few gradient steps. MAML++ [23] introduces multiple training refinements that improve stability, reduce hyperparameter sensitivity, and accelerate convergence. LEO [24] performs meta-learning in a compact latent space by learning a data-dependent generative representation, decoupled from high-dimensional parameter updates. ANIL [25] shows that feature reuse is central to performance and simplifies adaptation by restricting inner-loop updates to the task-specific classification head. UNICORN-MAML [26] mitigates evaluation instability from label permutations by meta-training a universal vector to initialize all classification head weights. Maxmin-MAML [27] adds inverted regularization at the inner level to reduce gradient variance and prevent overfitting. LA-PID-MAML [28] incorporates proportional–integral–derivative control to adapt layer-wise optimization gains and improve cross-domain generalization. The main advantage of gradient-based methods is their strong dynamic representation capacity, since task-specific updates can tailor features to domain shift. Their main limitation is optimization instability when randomly initialized classification heads are adapted from very few examples, which can distort early gradients and increase the hyperparameter tuning burden.

### 2.3. Integration of Metric and Gradient Meta-Learning

Proto-MAML [18] is the most representative attempt to connect metric learning and gradient-based meta-learning. Its original objective, however, is primarily to make the linear classifier compatible with tasks containing different numbers of classes. In practice, Proto-MAML uses class prototypes only to initialize a standard linear classification head, while the subsequent adaptation process remains largely identical to Model-Agnostic Meta-Learning. Consequently, it does not fully combine the core strengths of the two paradigms. In contrast, the proposed M^2^AML framework is designed to integrate the simple inductive bias of Prototypical Networks with the task adaptation capacity of Model-Agnostic Meta-Learning. The key structural difference from Proto-MAML is the complete removal of the parameterized linear classifier. The inner loop uses a pure Prototypical Network style update based on geometric probabilities, whereas the outer loop follows the Model-Agnostic Meta-Learning strategy to update meta-parameters globally. M^2^AML therefore preserves adaptation across variable-way tasks while providing better performance, faster training convergence, and simpler hyperparameter configuration.

## 3. Method

This section outlines the theoretical foundation and algorithmic pipeline of M^2^AML. We first formalize the few-shot classification problem and review relevant meta-learning paradigms. Finally, we detail the complete M^2^AML framework, including its unified adaptation procedure, self-exclusive prototype generalization, and batch normalization stabilization strategies.

### 3.1. Problem Formulation

**Problem setup.** Few-shot learning algorithms aim to establish robust generalization capabilities on novel categories using extremely limited labeled data. This learning paradigm operates on a comprehensive dataset D partitioned into three mutually exclusive subsets based on category labels. These subsets comprise a training dataset $D_{train}$, a validation dataset $D_{val}$, and a testing dataset $D_{test}$. The category spaces across these three subsets remain strictly disjoint. Instead of executing conventional batch optimization directly on individual samples, the meta-learning framework constructs a distribution of isolated classification tasks. During the optimization phase, the algorithm samples a sequence of training tasks $T_{train}$ exclusively from $D_{train}$ to optimize the model parameters. The framework subsequently evaluates the trained model by sampling evaluation tasks $T_{test}$ from $D_{test}$. Each individual task $T_{i}$ drawn from this distribution follows a rigorous episodic structure designed to simulate the few-shot evaluation environment. This structure partitions the sampled task data into a support set $S_{i}$ designated for task-specific parameter adaptation and a query set $Q_{i}$ reserved for objective evaluation.

**Standard *N*-way *K*-shot task.** Under the standard *N*-way *K*-shot classification protocol, the algorithm samples exactly *N* distinct categories from the available class pool. For each selected category, the support set contains strictly *K* definitively annotated instances. The corresponding query set comprises a distinct collection of unlabeled instances drawn uniformly from these same *N* categories. The primary objective is to learn a functional mapping $f_{\theta }$, parameterized by weights θ, that successfully extracts deep representations from the restricted support set to accurately predict the categorical identities of the query set instances.

**Open-task configuration.** Beyond uniformly configured episodic training, the open-task configuration introduces a critical evaluation mechanism to assess the generalized robustness of the model. In a strictly matched episodic framework, the structural parameters remain identical across all training and evaluation phases. Conversely, the open-task setting establishes an anchor paradigm where the model uniformly undergoes meta-training under a fixed *N*-way *K*-shot configuration. During the subsequent inference phase, the evaluation protocol samples tasks conforming to a completely different *M*-way *L*-shot specification. The dimensions *M* and *L* inherently deviate from the original anchor values *N* and *K*. This systematic structural discrepancy demands that the representational mechanisms within the model natively generalize across variable category capacities and diverse sample quantities without requiring architectural modifications.

### 3.2. Meta-Learning Paradigms

**MAML.** The MAML framework [10] operates by discovering an optimal set of initial parameters that can achieve rapid adaptation to any new task through a limited number of gradient descent steps. During the essential inner loop phase, the model computes the classification loss $L_{S_{i}}\left(\theta \right)$ specifically on the support set and proceeds to update the original parameters to a set of task-specific tuned parameters $\theta_{i}^{\prime }$.

(1) $$\theta_{i}^{\prime }=\theta -\alpha \nabla_{\theta }L_{S_{i}}\left(\theta \right).$$

The hyperparameter α fundamentally governs the inner loop learning rate. Subsequently, the outer loop evaluates these newly adapted parameters $\theta_{i}^{\prime }$ directly on the respective query set $Q_{i}$ to calculate the meta-objective. The framework updates the universal global initialization θ by minimizing the aggregate query set losses across a sampled batch of tasks.

(2) $$\theta \leftarrow \theta -\beta \nabla_{\theta }\sum_{T_{i}}L_{Q_{i}}\left(\theta_{i}^{\prime }\right).$$

The hyperparameter β controls the outer loop meta-update learning rate. Evaluating the exact gradient for this outer loop update necessitates computing computationally expensive second-order derivatives. By applying the chain rule, expanding the true meta-gradient explicitly reveals the strict dependency on the initial parameters θ.

(3) $$\nabla_{\theta }L_{Q_{i}}\left(\theta_{i}^{\prime }\right)=\nabla_{\theta_{i}^{\prime }}L_{Q_{i}}\left(\theta_{i}^{\prime }\right)\cdot \nabla_{\theta }\theta_{i}^{\prime }=\nabla_{\theta_{i}^{\prime }}L_{Q_{i}}\left(\theta_{i}^{\prime }\right)\cdot \left(I-\alpha \nabla_{\theta }^{2}L_{S_{i}}\left(\theta \right)\right).$$

The matrix I represents the identity matrix, and $\nabla_{\theta }^{2}L_{S_{i}}\left(\theta \right)$ signifies the Hessian matrix of the support loss. Computing this Hessian matrix imposes severe memory overhead and fundamentally restricts scalable training efficiency. To circumvent these computational limitations, this study utilizes the first-order approximation configuration. This practical approximation mathematically ignores the complex higher-order derivative terms by assuming $\nabla_{\theta }\theta_{i}^{\prime }\approx I$. Consequently, the surrogate first-order meta-gradient simplifies entirely to the gradient computed solely with respect to the newly adapted parameters.

(4) $$\nabla_{\theta }L_{Q_{i}}\left(\theta_{i}^{\prime }\right)\approx \nabla_{\theta_{i}^{\prime }}L_{Q_{i}}\left(\theta_{i}^{\prime }\right).$$

This formulation treats the inner loop gradients as fixed constants during the backward pass computation and successfully accelerates the training process without substantially degrading the resulting empirical performance.

**ProtoNet.** ProtoNet [14] establishes an effective paradigm by learning a continuous metric space where instances belonging to the same semantic class tightly aggregate. Given a support set where raw samples belong to distinct classes, the backbone network generates a feature embedding vector z for every input image. The algorithm computes a representative prototype vector $p_{c}$ for each specific class *c* by calculating the geometric mean of the corresponding support feature embeddings.

(5) $$p_{c}=\frac{1}{K}\sum_{z_{i}\in S_{i,c}}z_{i}.$$

This mathematical operation explicitly aggregates the observed features into a robust representative cluster center. The classification probability of a query sample depends entirely on evaluating the geometric distances between the extracted query feature and all established prototypes. ProtoNet conventionally applies the squared Euclidean distance to map features into normalized probabilities via a softmax distribution.

(6) $$P\left(y=c|z\right)=\frac{\exp(-∥z-p_{c}{∥}^{2})}{\sum_{k}\exp(-∥z-p_{k}{∥}^{2})}.$$

This specific formulation inherently relies on static feature spaces for inference. The mechanism entirely lacks task-level parameter optimization capabilities when encountering completely novel class domains.

**Proto-MAML.** While the standard MAML architecture initializes all network parameters from a single globally learned coordinate, the Proto-MAML [18] framework introduces a structural variant that directly leverages previously established metric space representations. The framework formally demonstrates that evaluating the negative squared Euclidean distance shares exact functional equivalence with a baseline linear classification layer. The negative squared distance systematically algebraically expands.

(7) $$-∥z-p_{c}{∥}^{2}=-{∥z∥}^{2}+2z^{⊤}p_{c}-{∥p_{c}∥}^{2}.$$

Because the quadratic term $-{∥z∥}^{2}$ remains completely independent of the selected class category, its specific component fully cancels out within the softmax probability calculation. The resulting mathematical structure mirrors an exact standard affine transformation, establishing a logit $L_{c}$ for the respective class.

(8) $$L_{c}\left(z\right)=z^{⊤}\left(2p_{c}\right)-{∥p_{c}∥}^{2}.$$

Proto-MAML explicitly exploits this fundamental equivalence by utilizing the support set prototypes to calculate deterministic geometric starting values for the final fully connected layer. For any specific class *c*, the classification weight vector $W_{c}$ and the corresponding statistical bias $b_{c}$ are uniquely initialized according to the extracted metric components.

(9) $$W_{c}=2p_{c},\;b_{c}=-{∥p_{c}∥}^{2}.$$

Following this deterministic metric initialization, the standard inner optimization loop executes task-specific parameter fine-tuning to meticulously refine both the backbone feature representations and these newly initialized classification boundaries.

### 3.3. Proposed M^2^AML Framework

**Figure 1:**
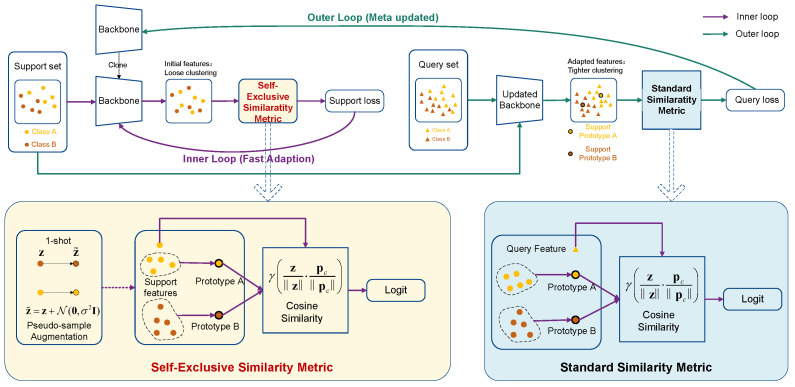
**The overall architectural framework of the proposed M^2^AML.** The top section illustrates the complete episodic meta-training pipeline, which excises parameterized classification layers in favor of geometric distance optimizations. The bottom sections detail the specific computational structures of the two distinct similarity components: the Self-Exclusive Similarity Metric utilized during inner loop task adaptation to prevent self-matching traps, and the Standard Similarity Metric employed for outer loop meta-updates.

**M^2^AML Algorithm Overview.** The overall pipeline of the proposed M^2^AML framework is illustrated in Figure 1 and detailed in Algorithm 1. Existing meta-learning paradigms face structural limitations: Prototypical Networks lack dynamic parameter adaptation, while MAML suffers from gradient instability due to randomly initialized classification heads. M^2^AML resolves this by completely eliminating parameterized classification layers from the adaptation loop.

During episodic training, the backbone first projects input images into a non-parametric metric space. In the inner loop, rather than adapting a classification head, the framework computes geometric similarities between support samples and class prototypes. The inner gradients directly fine-tune the feature extractor θ, forcing intra-class features to become highly compact and inter-class features distinctively separable. Subsequently, the outer loop evaluates the modified parameters $\theta_{i}^{\prime }$ on the query set, compelling query features to accurately converge toward their corresponding adjusted support prototypes.

Crucially, this architecture enables the use of a unified learning rate α for both inner loop task adaptation and outer loop meta-updates. Removing the randomly initialized classification layer naturally mitigates early-stage optimization shock, allowing synchronized optimization that eliminates complex hyperparameter grid searches. The definitive superiority of M^2^AML against contemporary state-of-the-art algorithms is comprehensively demonstrated in the empirical evaluations detailed in Section 4.2.
**Algorithm 1** M^2^AML Meta-Training Procedure**Require:** Distribution over tasks P(T), unified learning rate α, inner steps *T*, perturbation scale σ, boolean flags **robust_bn**, **isolate_bn****Ensure:** Optimized initial parameters θ and scaling factor γ  1:**Initialize**: Backbone parameters θ, learnable scale parameter γ  2:**while** not converged **do**  3:   Sample batch of tasks $T_{i}=\left(S_{i},Q_{i}\right)∼P\left(T\right)$ with labels $Y_{S_{i}},Y_{Q_{i}}$ $T_{i}$  4:   **for all** $T_{i}$ **do**  5:      // — Robust Batch Normalization Stabilization —  6:      **if robust_bn then**  7:        Execute gradient-free forward pass with task data ($S_{i}\cup Q_{i}$) to update global BN running statistics  8:      **end if**  9:      // Initialize task-specific parameters and BN buffers10:      $\theta_{i}^{\prime }\leftarrow \theta$11:      **if isolate_bn then**12:         $S_{BN}^{\prime }\leftarrow \mathrm{DeepCopy}\left(S_{BN}\right)$ // Isolate running statistical dict13:      **else**14:         $S_{BN}^{\prime }\leftarrow S_{BN}$15:      **end if**16:      // — Inner Loop (Task Adaptation) —17:      **for** k=1,…,T **do**18:         Extract support embeddings $z_{S_{i}}=f_{\theta_{i}^{\prime },S_{BN}^{\prime }}\left(S_{i}\right)$19:         Compute support logits $L_{S_{i}}=M_{\mathrm{self}\text{-}\mathrm{exc}}\left(z_{S_{i}},Y_{S_{i}},\gamma ,\sigma \right)$ (Algorithm 2)20:         Calculate inner loss: $L_{\mathrm{inner}}=\mathrm{CrossEntropy}\left(L_{S_{i}},Y_{S_{i}}\right)$21:         Update parameters: $\theta_{i}^{\prime }\leftarrow \theta_{i}^{\prime }-\alpha \nabla_{\theta_{i}^{\prime }}L_{\mathrm{inner}}$22:      **end for**23:      // — Outer Loop (Meta-Evaluation) —24:      Extract adapted support embeddings $z_{S_{i}}^{\prime }=f_{\theta_{i}^{\prime },S_{BN}^{\prime }}\left(S_{i}\right)$ and query embeddings $z_{Q_{i}}^{\prime }=f_{\theta_{i}^{\prime },S_{BN}^{\prime }}\left(Q_{i}\right)$25:      Compute query logits $L_{Q_{i}}=M_{\mathrm{standard}}\left(z_{S_{i}}^{\prime },Y_{S_{i}},z_{Q_{i}}^{\prime },\gamma \right)$ (Algorithm 3)26:      Calculate outer loss: $L_{\mathrm{outer}}^{\left(i\right)}=\mathrm{CrossEntropy}\left(L_{Q_{i}},Y_{Q_{i}}\right)$27:   **end for**28:   // Meta-Update (First-order approximation, utilizing unified learning rate α)29:   Update $\theta \leftarrow \theta -\alpha \sum_{T_{i}}\nabla_{\theta_{i}^{\prime }}L_{\mathrm{outer}}^{\left(i\right)}$30:   Update $\gamma \leftarrow \gamma -\alpha \nabla_{\gamma }\sum_{T_{i}}L_{\mathrm{outer}}^{\left(i\right)}$31:**end while**

**Self-Exclusive Prototype Generalization.** The framework explicitly differentiates the metric evaluations utilized in the inner and outer loops (Algorithms 2 and 3). During the outer loop meta-evaluation, standard prototype distances are effectively employed:

(10) $$P\left(y=c|x\right)=\frac{\exp(M_{\mathrm{standard}}\left(f_{\theta }\left(x\right),p_{c}\right))}{\sum_{k}\exp\left(M_{\mathrm{standard}}\left(f_{\theta }\left(x\right),p_{k}\right)\right)}.$$

Conversely, deploying standard aggregated prototypes directly inside the inner loop adaptation introduces a fatal self-matching optimization trap. Since support samples simultaneously act as evaluation queries and formulate cluster boundaries, calculating similarities without sample isolation minimizes self-inclusive distances rather than abstracting robust geometric metrics. To prevent this, internal computations switch to a self-exclusive framework (Algorithm 2) that rigorously masks local instances from their own class prototype calculation. Importantly, although this self-exclusive mechanism reduces the temporal prototype pool by exactly one instance, absolutely no training data is discarded from the optimization process. Let Ω represent the active feature distribution. The structural exclusion $\Omega_{c}\setminus \left\{z_{i}\right\}$ dynamically transitions the isolated sample into an active local query against its peers, structurally coerced into an exclusive prototype $p_{c}^{\left(i\right)}$. This internal pairing conceptually mirrors **Cross-Validation** executed directly within the episodic inner loop. By explicitly coercing the network to align each target sample toward the geometrically aggregated distribution of its distinct peers, the formulation drastically suppresses isolated feature memorization. Consequently, rather than inducing feature distortion in low-data regimes, this structural constraint actively enforces robust inter-sample semantic continuity, fundamentally maximizing gradient stability across the sparse manifold. Additionally, to avoid complete structural mathematical collapse under one-shot constraints, the algorithm systematically injects controlled isotropic Gaussian perturbations (ϵ∼N(0,I)), guaranteeing mathematically unbiased operational stability globally.

From a theoretical perspective, this injection transcends a simple engineering heuristic; it strictly operates as a **Manifold Smoothing Regularization** anchored in the principles of Vicinal Risk Minimization (VRM). Under extreme one-shot data scarcity, a strictly isolated spatial embedding $z_{i}$ mathematically collapses the class representation into a Dirac delta distribution $\delta (z-z_{i})$, completely lacking statistical variance. Evaluating a solitary sample against itself produces a constant similarity scaling metric of 1.0, which inherently results in an irreversible zero-gradient singularity. By systematically injecting independent isotropic noise ($\epsilon_{i}$), the local geometrical support of the highly sparse distribution is implicitly expanded into a continuous vicinal density:

(11) $$P_{\nu }\left(\tilde{z}|z_{i}\right)=N\left(\tilde{z};z_{i},\sigma^{2}I\right).$$

Consequently, treating the original embedding $z_{i}$ and its perturbed counterpart ${\tilde{z}}_{i}=z_{i}+\sigma \epsilon$, respectively, as a localized spatial query and a proxy prototype transforms the ill-posed deterministic trap into a stable neighborhood optimization constraint. The derivative of the similarity metric essentially bypasses the singular state, being explicitly directed by the orthogonal components of the perturbation vector instead:

(12) $$\nabla_{z_{i}}M_{\mathrm{self}\text{-}\mathrm{exc}}\left(z_{i},{\tilde{z}}_{i}\right)\approx \gamma \nabla_{z_{i}}\left(\frac{z_{i}^{⊤}\left(z_{i}+\sigma \epsilon \right)}{∥z_{i}∥∥z_{i}+\sigma \epsilon ∥}\right)\neq 0.$$

This mathematically robust manifold regularization forces the network to maintain intra-class semantic continuity even when interpolating across extreme data sparsity boundaries. Detailed ablation studies validating the efficacy of this self-exclusive mechanism are presented in Section 4.3.
**Algorithm 2** Self-Exclusive Similarity Metric ($M_{\mathrm{self}\text{-}\mathrm{exc}}$)**Require:** Support features $z_{S}$, Support labels $Y_{S}$, Scale γ, Perturbation scale σ**Ensure:** Unbiased support logits $L_{S}$  1:Initialize the active feature set $\Omega =\{\left(z_{i},y_{i}\right)\}$ from $z_{S}$ and $Y_{S}$.  2:**if** minimum shot count $K_{\min}==1$ **then**  3:   Sample independent perturbations $\epsilon_{i}∼N\left(0,I\right)$  4:   Augment feature set: $\Omega \leftarrow \Omega \cup \{\left(z_{i}+\sigma \epsilon_{i},y_{i}\right)\}$  5:**end if**  6:**for** each original support feature $z_{i}$ with label $y_{i}$ **do**  7:   **for** each class *c* **do**  8:     Let $\Omega_{c}$ denote all features in Ω belonging to class *c*.  9:     Compute exclusive prototype: $p_{c}^{\left(i\right)}=\frac{1}{|\Omega_{c}\setminus \left\{z_{i}\right\}|}\sum_{z\in \Omega_{c}\setminus \left\{z_{i}\right\}}z$10:     Compute logit: $L_{S}^{\left(i,c\right)}=\gamma \left(\frac{z_{i}^{⊤}p_{c}^{\left(i\right)}}{∥z_{i}∥∥p_{c}^{\left(i\right)}∥}\right)$11:   **end for**12:**end for**13:**return**$L_{S}$

**Batch Normalization Optimization Dynamics.** Tracking internal task-driven spatial iterations using standard batch sizes heavily degrades global normalization statistics. M^2^AML natively mitigates this via a synergistic integration of a **robust_bn** stabilization mechanism combined with an **isolate_bn** operational sequence (Algorithm 1).

Inspired by MAML++ [23], the **isolate_bn** operation explicitly creates a deep copy of the global Batch Normalization (BN) tracking dictionary strictly for the inner loop. This isolated computational buffer stops rapid task-driven feature distortions from permanently corrupting broader backbone standardizations. However, absolute inner loop isolation without an alternative update mechanism causes global BN stats to stably stagnate, fundamentally degrading evaluation performance.

To resolve this tracking paradox, the framework enforces a **robust_bn** stabilization phase before inner optimization. The network executes a standalone gradient-free forward pass over the entire aggregated task data ($S_{i}\cup Q_{i}$) to accurately pre-calibrate global population means and variance tensors. Subsequently, the **isolate_bn** mechanism is triggered, copying these freshly calibrated metrics internally for local evaluations. This synergistic approach prevents sequential statistical corruption while dynamically refreshing normalization profiles. It is critical to distinguish the original contribution of this synergistic strategy from prior gradient-based literature. While the **isolate_bn** concept conceptually originates from MAML++ [23], our proposed **robust_bn** mechanism is a strictly novel structural intervention designed explicitly for parameter-free metric architectures. In standard MAML frameworks, uncalibrated BN statistics can be partially absorbed and organically corrected by the dynamically updated weights of their parameterized linear classifiers. In stark contrast, M^2^AML completely eliminates this layer, relying exclusively on rigid geometric distance evaluations. Consequently, any local statistical distortion directly induces irreversible spatial warping within the metric manifold. By independently executing a prior gradient-free calibration, the original **robust_bn** phase acts not simply as a borrowed optimization stabilizer, but as a foundational geometric prerequisite that enforces strictly unbiased metric calculations under extreme data sparsity. Comprehensive ablation studies analyzing the precise impact of this batch normalization strategy are provided in Section 4.3.
**Algorithm 3** Standard Similarity Metric ($M_{\mathrm{standard}}$)**Require:** Support features $z_{S}$, Support labels $Y_{S}$, Query features $z_{Q}$, Scale γ**Ensure:** Query logits $L_{Q}$  1:**for** each class *c* **do**  2:   Let $S_{c}$ denote all features in $z_{S}$ belonging to class *c*.  3:   Compute standard prototype: $p_{c}=\frac{1}{|S_{c}|}\sum_{z\in S_{c}}z$  4:**end for**  5:**for** each query feature $z_{j}\in z_{Q}$ **do**  6:   **for** each class *c* **do**  7:     Compute logit: $L_{Q}^{\left(j,c\right)}=\gamma \left(\frac{z_{j}^{⊤}p_{c}}{∥z_{j}∥∥p_{c}∥}\right)$  8:   **end for**  9:**end for**10:**return** $L_{Q}$

### 3.4. Theoretical Divergence from Proto-MAML

As summarized in Table 2, the fundamental structural difference between Proto-MAML and M^2^AML lies in the retention versus complete excision of the parameterized linear classification layer. This architectural distinction directly dictates different optimization targets, learning rate strategies, and ultimately, distinct gradient propagation mechanisms.

While Proto-MAML elegantly applies metric concepts for initialization, it preserves the linear classification head (weights W and bias b) during inner-loop adaptation. Consequently, the gradient flow to the backbone parameters θ is inherently modulated by the dynamically updating weights of the linear classifier:

(13) $$\nabla_{\theta }L_{\mathrm{Proto}\text{-}\mathrm{MAML}}\propto {\left(\frac{\partial f_{\theta }\left(x\right)}{\partial \theta }\right)}^{⊤}W^{⊤}\nabla_{Wf_{\theta }\left(x\right)+b}L.$$

Because the optimization effort is shared between the classification head and the feature extractor, adapting the backbone’s spatial representations can be more challenging under extreme few-shot constraints where data points are scarce.

In contrast, our proposed M^2^AML framework completely removes the parameterized classifier from the adaptation sequence. The inner-loop fine-tuning relies purely on geometric similarities between the target features and dynamically aggregated class prototypes. This parameter-free metric optimization ensures that the adaptation gradients directly penalize the geometric distances within the embedding space without intermediary modulation:

(14) $$\nabla_{\theta }L_{M^{2}\mathrm{AML}}\propto \nabla_{\theta }M_{\mathrm{standard}}\left(f_{\theta }\left(x\right),p_{c}\right).$$

By strictly enforcing this direct metric update, M^2^AML focuses the entire optimization capacity on refining the feature manifold itself. This mechanistic streamlining naturally stabilizes the gradient trajectory, thereby enabling perfectly synchronized inner and outer learning rates.

## 4. Experiments

### 4.1. Datasets and Implementation Details

The evaluation utilizes three standard benchmark datasets. The ***mini*****-ImageNet** [19] dataset contains 100 classes with 600 images per class, resized to an 84 by 84 pixel resolution. The data partitioning exclusively assigns 64 classes for meta-training, 16 classes for meta-validation, and 20 classes for meta-testing. The ***tiered*****-ImageNet** [29] dataset presents a larger scale benchmark containing 779,165 images distributed across 608 categories, grouped into 34 semantic super-classes. To enforce substantial domain variation across phases, the evaluation protocol divides these structural super-classes directly into 20 for meta-training, 6 for meta-validation, and 8 for meta-testing. The **CIFAR-FS** [30] dataset adapts a pool of 100 classes composed of 32 by 32 pixel resolution images into an identical distribution, allocating 64 classes for training, 16 for validation, and 20 for evaluation.

The experiments employ a pre-trained ResNet-12 backbone and standard statistical normalization without complex data augmentation. The evaluation setup adopts a five-way one-shot configuration containing 15 queries per class. The meta-training process spans 20 epochs and sequences 500 episodes per epoch under a fixed random seed of 2025. The inner adaptation strictly executes five iterations using a self-exclusive cosine similarity metric augmented by a 0.01 jitter perturbation. The mechanism features robust batch normalization stabilization alongside corresponding isolated tracking statistics. The optimization utilizes a unified learning rate of 0.001 across both the inner adaptation and the outer first-order meta-update. The Nesterov Stochastic Gradient Descent optimizer operates with a momentum factor of 0.9 and a weight decay of 0.0005. The framework incorporates a cosine annealing learning rate scheduler starting with a five-epoch warmup phase. The similarity scalar is initialized at 10.0 for dynamic backpropagation without applying any label smoothing. The best model selected from single epoch validation evaluations undergoes testing across 2000 independent episodes to report the mean accuracy and the 95% confidence interval.

### 4.2. Main Results

**Comparative Evaluation against State-of-the-Art Algorithms.** The comprehensive performance evaluation presented in Table 3 and Table 4 systematically benchmarks the proposed M^2^AML framework against an extensive array of established few-shot classification algorithms previously documented in the literature. The empirical evidence demonstrates that the proposed architecture consistently establishes newly elevated performance standards irrespective of the spatial resolution or domain complexity. Operating within the *mini*-ImageNet one-shot configuration, the method achieves a definitive expected accuracy of 67.20%. This specific margin decisively surpasses robust existing models previously maintaining the performance ceiling. Expanding the evaluation to the substantially more complex *tiered*-ImageNet protocol reinforces this superiority by capturing a robust 71.87% accuracy under one-shot constraints and a solid 86.29% evaluation metric under five-shot conditions. Furthermore, translating the rigorous benchmarking protocol onto the CIFAR-FS dataset reveals an exact 76.61% classification mean within the isolated one-shot scenario. This specific computational result mathematically exceeds the highest performing prior comparative standard TPMN, which achieves exactly 75.5%, while the corresponding five-shot test configuration validates an output saturation accuracy reaching exactly 87.32%.

**Statistical Significance Analysis.** To address concerns regarding whether the performance improvements over recent powerful baselines emerge from marginal random test variance, we conducted a rigorous independent two-sample Welch’s *t*-test. As evaluation protocols in prior literature utilize varying test episode lengths—specifically, our method and RENet utilize N=2000, TPMN utilizes N=1000, while robust baselines like FEAT and FRN sample *N* = 10,000 episodes—this test accounts for unequal sample sizes and differing sample variances. The standard error (SE) is extrapolated explicitly using the formally reported 95% confidence intervals (SE≈CI/1.96).

The resulting empirical evidence, completely and transparently summarized in Table 5, conclusively proves that the majority of our numerical gains structurally exceed randomized evaluation variance (p<0.05). While highly saturated five-shot test boundaries (e.g., versus FRN on *mini*-ImageNet or TPMN on CIFAR-FS) predictably yield statistical ties, the fundamentally significant dominance across highly constrained one-shot evaluations explicitly verifies the optimization superiority and robust generalization capacity generated by omitting the parametric classification layer.

**Figure 2:**
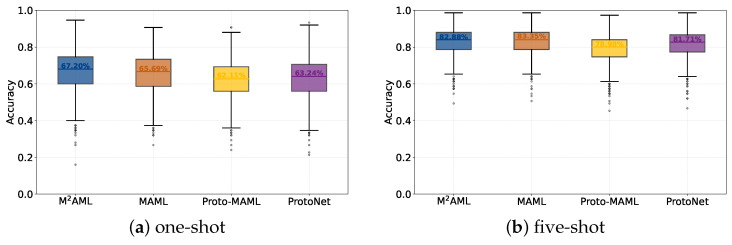
**Classification accuracy distributions on** ***mini*****-ImageNet.** All results are obtained from the same 2000 test tasks.

**Rigorous Baseline Verification and Statistical Stability Analysis.** To ensure an absolutely rigorous comparative analysis, avoiding implementation biases, the evaluation meticulously contrasts the M^2^AML architecture against the newly reproduced baseline models, including MAML, ProtoNet, and Proto-MAML. This targeted comparison strictly enforces utilizing identical pre-trained network backbones, entirely equivalent optimization algorithms, and comprehensively matched structural hyperparameters. The statistical distributions visualized in Figure 2 conclusively justify the algorithmic superiority by demonstrating a noticeably elevated median accuracy, accompanied by a tightly restricted performance variance across 2000 evaluation tasks. This definitive operational advantage directly originates from entirely removing the classical parameterized classification head and substituting the evaluation sequence specifically with an unbiased self-exclusive metric optimization phase. Conventional gradient-based structural models inherently suffer from severe gradient volatility induced by rapidly updating randomly initialized classification layers utilizing extremely sparse support sets. By executing task-specific fine-tuning exclusively through continuous geometric similarity calculations, the newly proposed M^2^AML framework systematically eliminates this initial optimization shock. This architectural refinement subsequently stabilizes the foundational gradient trajectory, naturally permitting the optimization process to achieve fundamentally deeper feature separation and consistently superior inference reliability.

**Figure 3:**
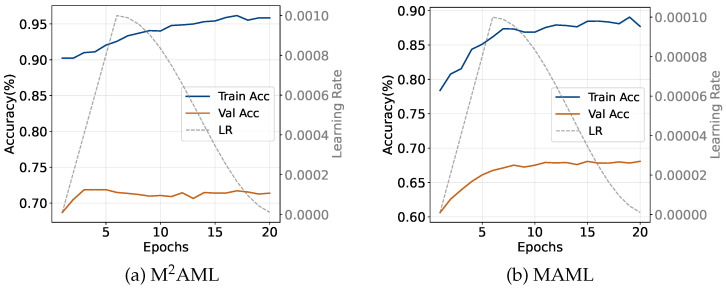
**Meta-training curves on** ***mini*****-ImageNet (one-shot).** The left vertical axis represents the classification accuracy, and the right vertical axis denotes the learning rate.

**Operational Efficiency and Optimization Advantages.** The introduced framework establishes several critical operational advantages, specifically distinguishing the architecture from established baseline algorithms. When compared to MAML, the proposed method demonstrates three primary structural improvements. First, the architecture achieves a *completely unified learning rate configuration*. While conventional gradient methods require distinct hyperparameters for internal adaptation and external updates, the proposed framework securely matches the inner and outer optimization step sizes. The algorithm strictly utilizes a constant learning rate of 0.001 for one-shot evaluations and 0.0015 for five-shot configurations. Second, the structural design guarantees *accelerated functional task adaptation*. By eliminating the parameterized classification layer, the network requires substantially fewer inner loop iterations to establish robust decision boundaries. The methodology successfully adapts using exactly five optimal steps during one-shot evaluations and 10 steps under five-shot conditions, whereas MAML mandatorily demands 10 and 20 corresponding iterations. Third, this configuration directly produces *profoundly faster global meta-training convergence*. As explicitly visualized within the optimization trajectories in Figure 3, the proposed method converges rapidly during the initial training phases. Standard MAML architectures endure severe convergence delays because they must repeatedly reconstruct novel linear boundary parameters from randomized states for every sampled task. Importantly, this specific design choice inherently introduces a structural trade-off regarding the theoretical adaptation capacity of the model. By excising the parameterized linear classification head, the network intentionally forfeits the ability to perform arbitrary affine transformations or construct highly complex, distorted decision boundaries during inner-loop updates. While standard MAML theoretically possesses a higher adaptation capacity via this flexible linear layer, under extreme few-shot constraints (especially one-shot configurations), this excessive parametric capacity heavily provokes severe overfitting and gradient volatility. M^2^AML explicitly trades this superficial linear flexibility for profound spatial stabilization. By restricting adaptation exclusively to the backbone feature extractor through pure geometric similarities, the network is algorithmically forced to cluster the underlying representation manifold into a strictly linearly separable metric space. Consequently, what the model sacrifices in arbitrary boundary elasticity, it overwhelmingly regains in gradient reliability and robust resistance to sparse-data overfitting.

**Figure 4:**
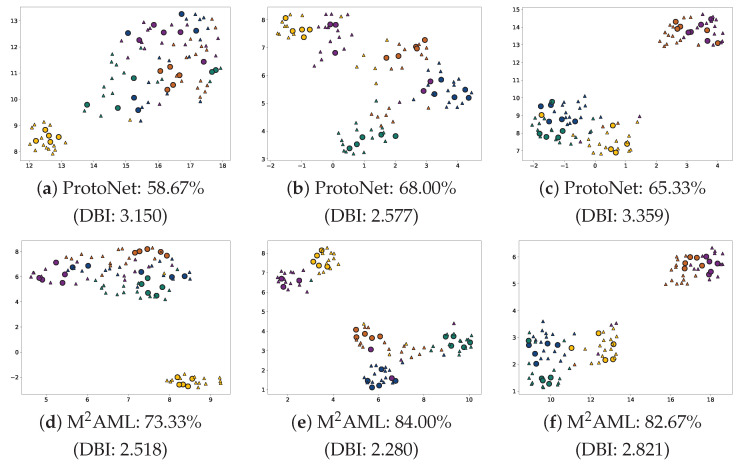
**UMAP. [44] Feature Distributions on** ***mini*****-ImageNet.** The upper sequence (**a**–**c**) illustrates independent five-way five-shot evaluation tasks executed by the ProtoNet baseline. The lower sequence (**d**–**f**) visualizes the identical task configurations optimally processed through the proposed M^2^AML architecture. To quantitatively assess cluster separability, each subfigure reports the Davies-Bouldin Index (DBI), a clustering metric where lower values indicate better separation. Distinct colors identify different classes. The circle marker ∘ denotes support instances. The triangle marker ▵ indicates query instances.

When compared to purely static metric approaches like ProtoNet, the targeted inner loop generates major representational benefits alongside a managed computational trade-off. The fundamental advantage is *superior feature space discrimination*. As demonstrated by the feature distribution visualizations in Figure 4, the explicit task adaptation mechanism dynamically forces intra-class features to become highly compact while ensuring inter-class clusters remain distinctly separable. This continuous spatial optimization inherently leads to a *moderately increased computational overhead*. To quantify this operational trade-off, Table 6 evaluates the computational costs on a single NVIDIA RTX 3090 GPU. While M^2^AML naturally requires more computation than parameter-free ProtoNet due to its inner-loop adaptations, excising the linear classification head enables it to achieve 16% faster training and 35% lower inference latency compared to standard MAML, all while maintaining an identical peak memory footprint (<5.0 GB).

Finally, comparing the framework directly against the hybrid capabilities of Proto-MAML validates the systematic removal of the linear classification layer. The empirical distributions detailed in Table 3 and Table 4 solidly confirm *consistently elevated classification performance*. As detailed in the prior methodological analysis sections, substituting linear definitions with geometric initialization effectively forces Proto-MAML to operate practically identically to the MAML optimization sequence. The newly proposed mechanism avoids these parameterized bottlenecks completely, structurally proving that continuous geometric similarity evaluations functionally exceed standard fine-tuned linear transformations.

### 4.3. Ablation Studies

**Batch normalization stabilization dynamics.** Traditional meta-learning architectures natively struggle with batch normalization statistics due to continuous data distribution shifts within the inner loop. The experiments presented in Table 7 systematically isolate the contributions of the *robust* calibration phase and the completely *isolated* tracking configuration. For the one-shot configuration, the support set contains extremely limited samples. Because this sparse data cannot generate reliable normalization statistics independently, merely isolating the batch normalization buffers proves insufficient. In this specific low-data regime, combining the robust setup with strict isolation properly updates the statistics utilizing the combined task data before sealing the internal adaptation process.

Conversely, the five-shot configuration expands the support set to 25 samples. This increased capacity provides enough statistical stability for the inner loop to update batch normalization structures reliably. When sufficient samples exist, introducing the robust calibration mechanism actually degrades performance because pre-calibrating with query samples unnecessarily distorts the robust intra-class boundaries already established by the adequate support set. Therefore, simply applying strict isolation without the robust mechanism generates optimal results in the five-shot setting. This trend remains consistent across both standard MAML structures and the proposed M^2^AML framework. The unadjusted raw geometric task adaptation in M^2^AML otherwise struggles with statistical divergence without these stabilization measures.

Finally, Table 8 verifies that the batch normalization updating protocol varies fundamentally between training and testing environments. During the meta-training phase, continuously updating statistics reflects the dynamic shifts needed to learn generalized parameters. However, during the meta-testing phase, the global network parameters and tracking statistics are already completely stable. Dynamic statistical updates during evaluation introduce unwanted mathematical noise into a properly stabilized representation domain. Therefore, fixing the explicit batch normalization parameters strictly during testing preserves the aligned feature mapping and inherently maximizes the resulting accuracy across both baseline and geometric architectures.

**Self-exclusive inner loop adaptation.** The structural decision to implement a self-exclusive geometric prototype evaluation during the internal task adaptation constitutes a critical mathematical component of the proposed framework. The experimental baseline explicitly compares this proposed mechanism against a standard inclusive prototype calculation strategy. Within a conventional inclusive setup, the framework aggregates all support features into a single cluster centroid $p_{c}=\frac{1}{K}\sum_{i=1}^{K}z_{i}$. When the optimization engine evaluates the specific logit for a target support sample $z_{i}$ exactly towards its own class prototype, the resulting distance metric implicitly embeds the strict identity vector itself. For a one-shot classification task where K=1, the evaluated logit essentially computes the spatial similarity between the sample feature and its exact geometric duplicate.

(15) $$L_{c}^{\left(i\right)}=\gamma \left(\frac{z_{i}^{⊤}z_{i}}{∥z_{i}∥∥z_{i}∥}\right)=\gamma .$$

This structural redundancy instantly maximizes the positive class probability allocation. Although the model outputs a categorical classification accuracy perfectly reaching 100% on the local support set during the initial forward pass, the underlying mathematical gradients strictly refuse to vanish. The corresponding loss function still applies an active optimization signal explicitly driving the unscaled dot product magnitude toward theoretical infinity while simultaneously violently penalizing the logits corresponding to the alternative classes. This specific gradient behavior essentially optimizes a continuous structural scaling magnitude rather than forcing meaningful feature separation. The results presented in Table 9 consistently demonstrate that deploying this standard inclusive mapping strategy persistently degrades functional accuracy regardless of the batch normalization setup applied.

Substituting this mechanism with the stringent self-exclusive isolation entirely solves this paradox. By actively masking the evaluating sample $z_{i}$ securely from its target true class prototype calculation, the ensuing gradient vectors are strictly compelled to recognize the absolute underlying spatial distribution governing the remaining instances. Even operating strictly under demanding one-shot thresholds utilizing the mathematical perturbation setup, this forced relational evaluation successfully prevents artificial scaling domination. The empirical outputs validate this theoretical insight. Executing the self-exclusive mechanism consistently propels optimization capabilities substantially beyond all tested inclusive parameter combinations.

**Learning rate synchronization and functional adaptation step analysis.** Gradient-based meta-learning conventionally struggles with navigating intense optimization volatility. Standard frameworks typically rely on establishing distinct variable learning rates for internal adaptation and external meta-updates while mandating substantial integration steps strictly to achieve convergence. The experimental evaluations presented in Table 10 and Table 11 systematically assess how structural modification impacts these specific hyperparameters under the proposed M^2^AML structure. A fundamental configuration shift emerges from the empirical performance data. Unlike baseline methodologies, the proposed framework consistently reaches its absolute classification ceiling precisely when the internal learning rate identically matches the global unified external optimization schedule. Maintaining a synchronized updating velocity prevents asynchronous optimization paths from creating conflicting representational shifts during the meta-update phase.

Furthermore, analyzing the internal iteration thresholds reveals a distinct acceleration in necessary adaptation sequences. The data demonstrates that executing exactly five operational inner loop steps perfectly extracts robust spatial boundaries for a one-shot configuration. Correspondingly, increasing the sample density under the five-shot configuration requires merely 10 steps to fully synthesize a mature decision manifold. Substantially escalating internal iterations uniformly triggers structural degradation and classification accuracy depression. This accelerated convergence dynamic inherently stems directly from substituting the conventional randomly initialized linear classifier completely with a rigid geometric metric baseline. During standard architecture optimization, massive quantities of initial gradient steps are fundamentally wasted entirely attempting to organize and orient the mathematically chaotic randomized head boundaries. The proposed geometric backbone circumvents this chaotic parameter initialization completely and natively permits immediate meaningful feature space orientation right from the initial internal optimization step. The synchronized learning configurations paired with compressed iteration constraints conclusively provide a structurally optimized mathematical pathway enabling rapid, reliable task adaptation.

**Auxiliary hyperparameter configurations. **Table 12 systematically evaluates the remaining critical structural hyperparameters governing the proposed architecture under the one-shot configuration setting. Further confirming that these optimal values are not dataset-tailored heuristics, Table 13 independently verifies corresponding sensitivity on the CIFAR-FS benchmark, consistently demonstrating that γ=10 and σ=0.01 construct the optimal generalization manifold without requiring dataset-specific tuning. The initialization magnitude of the learnable scaling factor γ implicitly controls the initial softmax probability spread over the computed logits. Setting this scalar strictly at a value of 10 precisely balances gradient propagation limits. Lower scaling configurations structurally induce severely diluted classification probability distributions, resulting in flattened gradient signals. Conversely, setting excessively large initialization thresholds saturates the confidence probabilities instantly and triggers premature vanishing gradient phenomena during initial adaptation phases.

The precision jitter evaluation analyzes the effect of embedding artificial Gaussian mathematical noise into support samples specifically directed to artificially satisfy the structural limitations of one-shot self-isolation. Integrating a minimal perturbation variance scaling of exactly 0.01 provides a stable spatial distinction sufficient to bypass the zero-distance trap while perfectly retaining foundational true spatial semantics. Larger magnitude configurations obscure structural feature semantics and cause overlapping representational clusters.

Standard label smoothing generally reduces structural overfitting behaviors for supervised classification pipelines via regularizing continuous target spaces. Applying classical categorical label smoothing to the M^2^AML framework progressively degrades the resulting geometric optimization vectors. Distributing probability constraints continuously across competing structural categories mathematically contradicts the fundamental goal of driving discrete spatial distances maximally far apart. The baseline probability vectors naturally guide optimal boundary separation, strictly executing optimization parameters utilizing hard categorical annotations representing 0.00 smoothing logic.

Finally, substituting the inherent evaluation metric space completely dictates overall classification potentiality. Formulating similarity evaluations, specifically manipulating standard L1 norms and generic Euclidean L2 norms, categorically suppresses ultimate operational capacity. The cosine similarity metric restricts projection logic entirely onto an idealized unit hypersphere domain. This continuous angle-bounded calculation specifically isolates structurally robust rotational feature separations independent of sheer magnitude variations. The mathematical geometry natively enforced by cosine similarity computations fundamentally unlocks the optimal representational mappings. To theoretically and empirically address the concern that purely metric methods often benefit from numeric scale, we clarify that scale is functionally decoupled rather than completely discarded in our architecture. The unbounded magnitudes of standard Euclidean distances (L2) routinely induce extreme gradient saturation when directly mapped to local softmax distributions, causing inner-loop adaptation to destabilize. By explicitly projecting features onto an idealized unit hypersphere via cosine similarity, we forcibly eliminate these chaotic norm disparities to ensure stable angular decision boundaries. However, to preserve the necessary magnitude scaling required for effective softmax probability propagation, our framework systematically reintroduces a globally learnable probability scalar (γ). As explicitly corroborated by the distance metric ablations in Table 12, substituting this decoupled metric approach with standard Euclidean L2 distances (which natively preserve unconstrained magnitude) drastically degrades performance. Concurrently, our scaling ablations demonstrate that insufficient scalar initializations severely flatten the probability distributions, validating the necessity of the artificial scale constraint. This dual algorithmic design—structurally separating rotational directionality (Cosine) from probability scaling (γ)—is therefore empirically proven to construct the optimally stable metric environment.

## 5. Conclusions

This study introduced Metric-based Model-Agnostic Meta-Learning, a unified framework that fundamentally eliminates the optimization instability inherent in standard few-shot architectures. By completely removing the parameterized classification head and integrating an unbiased self-exclusive geometric metric, the methodology enabled perfectly synchronized optimization schedules and substantially accelerated task adaptation while achieving state-of-the-art accuracy across standardized benchmarks. Quantitatively, M^2^AML delivers absolute classification improvements ranging from 0.1% to 2.1% over established leading models while maintaining identical baseline structural complexity. Furthermore, it operates 16% faster during meta-training and reduces inference latency by approximately 35% compared to standard gradient-based baselines. Future research will focus on extending this geometric adaptation mechanism to cross-domain few-shot environments where severe distribution shifts inherently constrain global parameter initializations. Additionally, integrating optimal transport structures to model fine-grained local semantic alignments presents a promising trajectory for further expanding representational generalizability.

## Figures and Tables

**Table 1:** **Comparison of Meta-Learning Algorithms.** Performance on *mini*-ImageNet with ResNet-12. For inner learning rates, Separate indicates distinct configurations, Unified shares the exact outer rate, and None lacks inner adaptation.

Method	Meta-Training	Inner LR	Inference	One-Shot	Five-Shot
MAML	Slow	Separate	Slow	65.69 ± 0.44	83.41 ± 0.29
ProtoNet	Fast	None	Fast	63.24 ± 0.46	81.71 ± 0.30
M^2^AML	Moderate	Unified	Moderate	67.20 ± 0.45	82.88 ± 0.30

**Table 2:** **Fundamental structural differences between Proto-MAML and M^2^AML**.

Structural Mechanism	Proto-MAML	M^2^AML (Ours)
Inner-loop Classifier	Parameterized Linear Layer	Parameter-free Metric
Learnable Parameters	{θ,W,b}	Core Backbone {θ}
Gradient Flow to Backbone	Modulated by W	Direct Distance Derivatives
Optimization Target	Linear Boundary and Features	Pure Geometric Space
Learning Rate Strategy	Disjoint (α≠β)	Synchronized (α=β)

**Table 3:** **Classification Performance on** ***mini*****-ImageNet and** ***tiered*****-ImageNet.** Baseline results for MAML, ProtoNet, and Proto-MAML reflect local reproductions. All remaining metrics are directly sourced from published benchmark records. Hyphens indicate unreported values. Bold values emphasize the highest performance. Underlined values designate the second-highest performance.

Method	Backbone	Mini One-Shot	Mini Five-Shot	Tiered One-Shot	Tiered Five-Shot
MatchingNet [19]	Conv4	46.6	60.0	–	–
ProtoNet [14]	Conv4	49.42 ± 0.78	68.20 ± 0.66	–	–
TADAM [20]	ResNet-12	58.5 ± 0.3	76.7 ± 0.3	–	–
Semi-ProtoNet [29]	ResNet-12	50.41 ± 0.31	64.39 ± 0.24	52.39 ± 0.44	69.88 ± 0.20
LEO [24]	WRN-28-10	61.76 ± 0.08	77.59 ± 0.12	66.33 ± 0.05	81.44 ± 0.09
R2D2 [30]	Conv4	51.9 ± 0.2	68.7 ± 0.2	–	–
Baseline++ [31]	ResNet-18	51.87 ± 0.77	75.68 ± 0.63	–	–
MetaOptNet [32]	ResNet-12	64.09 ± 0.62	80.00 ± 0.45	65.81 ± 0.74	81.75 ± 0.53
SimpleShot [33]	ResNet-18	62.85 ± 0.20	80.02 ± 0.14	69.09 ± 0.22	84.58 ± 0.16
CC+rot [34]	WRN-28-10	64.03 ± 0.46	80.68 ± 0.33	70.53 ± 0.51	84.98 ± 0.36
RFS-simple [35]	ResNet-12	62.02 ± 0.63	79.64 ± 0.44	69.74 ± 0.72	84.41 ± 0.55
DeepEMD [21]	ResNet-12	65.91 ± 0.82	82.41 ± 0.56	71.16 ± 0.87	86.03 ± 0.58
FEAT [16]	ResNet-12	66.78 ± 0.20	82.05 ± 0.14	70.80 ± 0.23	84.79 ± 0.16
Meta-Baseline [17]	ResNet-12	63.17 ± 0.23	79.26 ± 0.17	68.62 ± 0.27	83.74 ± 0.18
FRN [36]	ResNet-12	66.45 ± 0.19	82.83 ± 0.13	71.16 ± 0.22	86.01 ± 0.15
CG [37]	ResNet-12	63.56 ± 0.20	79.13 ± 0.14	71.66 ± 0.23	85.50 ± 0.15
MetaQDA [38]	ResNet-18	65.12 ± 0.66	80.98 ± 0.75	69.97 ± 0.52	85.51 ± 0.58
MAML [26]	ResNet-12	64.42 ± 0.20	**83.44 ± 0.14**	65.72 ± 0.20	84.37 ± 0.16
LDA+L2-norm [39]	ResNet-12	60.29 ± 0.80	75.99 ± 0.66	64.16 ± 0.90	81.96 ± 0.65
Meta-AdaM [13]	ResNet-12	59.89 ± 0.49	77.92 ± 0.43	65.31 ± 0.48	85.24 ± 0.35
Minimax-MAML++ [27]	Conv4	53.28 ± 0.35	71.70 ± 0.23	–	–
LA-PID-MAML [28]	ResNet-12	63.29 ± 0.48	79.18 ± 0.43	64.77 ± 0.47	82.59 ± 0.37
NIW-Meta [40]	ResNet-18	65.49 ± 0.56	81.71 ± 0.17	70.52 ± 0.19	85.83 ± 0.17
SSCF [41]	VGGSNN	60.97 ± 0.45	75.61 ± 0.34	–	–
MAML (Reproduced)	ResNet-12	65.69 ± 0.44	83.41 ± 0.29	69.78 ± 0.23	83.93 ± 0.19
ProtoNet (Reproduced)	ResNet-12	63.24 ± 0.46	81.71 ± 0.30	68.41 ± 0.23	85.47 ± 0.15
Proto-MAML (Reproduced)	ResNet-12	62.11 ± 0.45	78.98 ± 0.32	63.72 ± 0.23	83.57 ± 0.16
**M^2^AML (Ours)**	ResNet-12	**67.20 ± 0.45**	82.88 ± 0.30	**71.87 ± 0.23**	**86.29 ± 0.16**

**Table 4:** **Classification Performance on CIFAR-FS.** Baseline results for MAML, ProtoNet, and Proto-MAML reflect local reproductions. All remaining metrics are directly sourced from published benchmark records. Bold values emphasize the highest performance. Underlined values designate the second-highest performance.

Method	Backbone	CIFAR-FS One-Shot	CIFAR-FS Five-Shot
R2D2 [30]	Conv4	65.3 ± 0.2	78.3 ± 0.2
MetaOptNet [32]	ResNet-12	72.8 ± 0.7	85.0 ± 0.5
RFS-simple [35]	ResNet-12	71.5 ± 0.88	86.0 ± 0.5
CG [37]	ResNet-12	73.0 ± 0.7	85.8 ± 0.5
TPMN [42]	ResNet-12	75.5 ± 0.9	87.2 ± 0.6
RENet [43]	ResNet-12	74.51 ± 0.46	86.60 ± 0.32
LDA+L2-norm [39]	ResNet-12	65.57 ± 0.89	80.80 ± 0.68
LA-PID-MAML [28]	ResNet-12	71.44 ± 0.45	85.15 ± 0.35
MAML (Reproduced)	ResNet-12	71.67 ± 0.51	86.50 ± 0.35
ProtoNet (Reproduced)	ResNet-12	72.74 ± 0.48	86.79 ± 0.33
Proto-MAML (Reproduced)	ResNet-12	68.19 ± 0.49	83.99 ± 0.35
**M^2^AML (Ours)**	ResNet-12	**76.61 ± 0.48**	**87.32 ± 0.34**

**Table 5:** **Statistical Significance Test (Welch’s *t*-test) against Strongest Baselines.** Evaluation of the independent *t*-statistics and *p*-values across datasets. A p-value<0.05 functionally validates that the numerical gains are statistically significant. The complete analysis transparently includes both one-shot and saturated five-shot evaluations to objectively track optimization ceilings.

Dataset	Evaluation vs. Baseline (*N*)	Δ Accuracy	*t*-Statistic	*p*-Value	Conclusion (p<0.05)
*mini*-ImageNet	one-shot vs. FRN (10000)	+ 0.75%	3.010	0.0026	Highly Significant
	one-shot vs. FEAT (10000)	+ 0.42%	1.672	0.0945	Marginal
	five-shot vs. FRN (10000)	+ 0.05%	0.300	0.7641	Statistical Tie
	five-shot vs. FEAT (10000)	+ 0.83%	4.917	<0.0001	Highly Significant
*tiered*-ImageNet	one-shot vs. FRN (10000)	+ 0.71%	4.369	<0.0001	Highly Significant
	one-shot vs. FEAT (10000)	+ 1.07%	6.442	<0.0001	Highly Significant
	five-shot vs. FRN (10000)	+ 0.28%	2.496	0.0126	Significant
	five-shot vs. FEAT (10000)	+ 1.50%	12.95	<0.0001	Highly Significant
CIFAR-FS	one-shot vs. TPMN (1000)	+ 1.11%	2.133	0.0330	Significant
	one-shot vs. RENet (2000)	+ 2.10%	6.189	<0.0001	Highly Significant
	five-shot vs. TPMN (1000)	+ 0.12%	0.341	0.7331	Statistical Tie
	five-shot vs. RENet (2000)	+ 0.72%	3.021	0.0025	Highly Significant

**Table 6:** Cost comparison on *mini*-ImageNet (one-shot) using a single NVIDIA RTX 3090. Inference latency is averaged across 2000 episodes.

Method	Peak GPU Memory	FLOPs	Training Time	Inference Latency
ProtoNet	<5.0 GB	7.04 G	30 m	31.12 ms
MAML	<5.0 GB	7.07 G	61 m	148.73 ms
M^2^AML (Ours)	<5.0 GB	7.04 G	51 m	96.91 ms

**Table 7:** **Batch normalization strategy ablation on** ***mini*****-ImageNet.** The *robust* configuration merges support and query sets for a unified gradient-free forward pass to exclusively update running statistics. The *isolated* configuration completely prevents inner loop optimization steps from modifying the backbone running statistics. All experiments uniformly implement a 0.0001 learning rate alongside 10 inner loop iterations for the MAML baseline and 5 for the proposed M^2^AML framework across both one-shot and five-shot settings. Here, ✓ and × denote enabled and disabled settings, respectively. Bold values indicate the best performance within each method block.

Method	Robust	Isolated	One-Shot	Five-Shot
MAML	×	×	65.07 ± 0.45	83.18 ± 0.30
	×	✓	65.40 ± 0.44	**83.41 ± 0.29**
	✓	×	65.07 ± 0.45	83.18 ± 0.30
	✓	✓	**65.69 ± 0.44**	83.35 ± 0.29
M^2^AML	×	×	66.67 ± 0.45	81.88 ± 0.31
	×	✓	66.36 ± 0.45	**82.50 ± 0.30**
	✓	×	66.53 ± 0.44	81.81 ± 0.31
	✓	✓	**67.01 ± 0.45**	82.41 ± 0.30

**Table 8:** **Test phase batch normalization ablation on** ***mini*****-ImageNet.** The Fixed BN setting indicates that the batch normalization running statistics remain unupdated during evaluation. Here, ✓ and × denote fixed and unfixed BN settings, respectively.

Method	Fixed BN	One-Shot	Five-Shot
MAML	×	65.58 ± 0.45	83.42 ± 0.29
	✓	65.69 ± 0.44	83.41 ± 0.29
M^2^AML	×	66.61 ± 0.47	81.26 ± 0.31
	✓	67.01 ± 0.45	82.50 ± 0.30

**Table 9:** **Self-exclusive prototype calculation ablation on** ***mini*****-ImageNet.** The Full strategy constructs class prototypes utilizing all available support samples, including the evaluated instance. The Self-Exc strategy strictly masks the currently evaluated instance from its corresponding prototype calculation during the inner loop. Here, ✓ and × denote enabled and disabled settings, respectively. Bold values indicate the best performance.

Prototype Strategy	Robust	Isolate	One-Shot	Five-Shot
Full	×	×	65.39 ± 0.44	81.81 ± 0.30
	×	✓	65.91 ± 0.45	82.15 ± 0.30
	✓	×	65.29 ± 0.44	81.79 ± 0.30
	✓	✓	66.31 ± 0.44	81.65 ± 0.31
Self-Exc	✓	✓	**67.20 ± 0.45**	**82.50 ± 0.35**

**Table 10:** **Learning rate and inner step ablation on** ***mini*****-ImageNet (one-shot).** The equivalent symbol (=) designates when the inner adaptation learning rate strictly equals the unified external updating configuration. Bold values indicate the best performance.

Inner Iterations	LR	Inner LR	One-Shot
5	0.00005	=	66.75 ± 0.45
	0.0001	=	67.01 ± 0.45
	0.0005	=	66.68 ± 0.45
	0.001	0.001	**67.20 ± 0.45**
		0.01	66.76 ± 0.45
		0.05	66.16 ± 0.45
		0.1	63.62 ± 0.53
	0.0015	=	66.74 ± 0.45
	0.002	=	66.58 ± 0.46
10	0.0001	=	67.00 ± 0.45
	0.001	0.001	66.77 ± 0.45
		0.01	66.58 ± 0.46
		0.05	65.93 ± 0.46
		0.1	63.94 ± 0.54
	0.0015	=	66.65 ± 0.46

**Table 11:** **Learning rate and inner step ablation on** ***mini*****-ImageNet (five-shot).** The equivalent symbol (=) designates when the inner adaptation learning rate strictly equals the unified external updating configuration. Bold values indicate the best performance.

Inner Iterations	LR	Inner LR	Five-Shot
5	0.0001	=	82.50 ± 0.00
	0.0005	=	82.65 ± 0.30
	0.001	=	82.65 ± 0.30
10	0.00005	=	82.39 ± 0.30
	0.0001	=	82.50 ± 0.30
	0.0005	=	82.67 ± 0.30
	0.001	=	82.79 ± 0.30
	0.0015	0.0015	**82.88 ± 0.30**
		0.005	82.74 ± 0.30
		0.01	82.89 ± 0.30
		0.05	73.30 ± 0.62
		0.1	47.50 ± 0.48
20	0.0015	0.01	82.67 ± 0.31
	0.0015	0.005	82.78 ± 0.31

**Table 12:** **Additional Hyperparameter Ablation on** ***mini*****-ImageNet (one-shot).** The evaluation analyzes the impact of the initial scaling factor, mathematical jitter magnitude, categorical label smoothing, and geometric distance metric definitions on overall performance.

Component	Configuration	One-Shot
Scaling Factor	5	65.74 ± 0.46
	10	67.20 ± 0.45
	15	66.55 ± 0.45
Jitter	0.001	66.82 ± 0.46
	0.01	67.20 ± 0.45
	0.05	67.20 ± 0.45
Label Smoothing	0.00	67.20 ± 0.45
	0.10	67.05 ± 0.45
	0.15	66.69 ± 0.45
	0.20	66.45 ± 0.45
Distance Metric	L1	61.06 ± 0.46
	L2	59.72 ± 0.46
	Cosine	67.20 ± 0.45

**Table 13:** **Hyperparameter Sensitivity Analysis on CIFAR-FS (one-shot).** The optimal stabilization values structurally generalize across distinct datasets. Bold values indicate the best performance.

Hyperparameter	Value	Accuracy (%)
Perturbation Scale	0.001	75.81 ± 0.49
	0.01	**76.61 ± 0.48**
	0.05	76.19 ± 0.49
Scaling Factor	5	75.81 ± 0.49
	10	**76.61 ± 0.48**
	15	76.19 ± 0.49

## Data Availability

All datasets used in this paper are publicly accessible through the learn2learn library.
